# Draft Genome Assembly of *Colletotrichum chlorophyti*, a Pathogen of Herbaceous Plants

**DOI:** 10.1128/genomeA.01733-16

**Published:** 2017-03-09

**Authors:** P. Gan, M. Narusaka, A. Tsushima, Y. Narusaka, Y. Takano, K. Shirasu

**Affiliations:** a RIKEN Center for Sustainable Resource Science, Kanagawa, Japan; b Research Institute of Biological Sciences, Okayama, Japan; c Graduate School of Science, University of Tokyo, Tokyo, Japan; d Graduate School of Agriculture, Kyoto University, Kyoto, Japan

## Abstract

*Colletotrichum chlorophyti* is a fungal pathogen that infects various herbaceous plants, including crops such as legumes, tomato, and soybean. Here, we present the genome of *C. chlorophyti* NTL11, isolated from tomato. Analysis of this genome will allow a clearer understanding of the molecular mechanisms underlying fungal host range and pathogenicity.

## GENOME ANNOUNCEMENT

*Colletotrichum* spp. comprise a group of diverse fungi, many of which are pathogens of agriculturally important plants. Among these, *C. chlorophyti* has been reported to associate with a variety of herbaceous plant species, including important crop plants such as legumes (1), tomato, and soybean (2). Infections have been reported to occur on leaves, as well as in seeds. Phylogenetic analysis has revealed that *C. chlorophyti* does not belong to any of the major species complexes identified in the *Colletotrichum* genus to date whose members have previously been sequenced (3), although it is closely related to *C. phaseolorum*, which is also a known pathogen of soybean. Thus, the genome sequence of *C. chlorophyti* will be useful not only by providing information of an agricultural pathogen but also for genus-wide studies analyzing *Colletotrichum* diversity and host range. In this study, we present the draft genome sequence of *C. chlorophyti* strain NTL11, which was isolated from infected tomato leaves.

Genomic DNA was isolated from hyphae grown *in vitro* and purified using the Genomic-tip 100/G kit (QIAgen) following the protocol described for the 1000 Fungal Genomes Project. Two 100-bp paired-end libraries with approximately 150-bp and 500-bp insert sizes were prepared using the TruSeq DNA PCR-Free library preparation kit and sequenced using the Illumina HiSeq 2500 platform (RIKEN OSC) with 54× coverage. Reads were trimmed using Trimmomatic version 0.33 (4). The acquired reads were assembled using SOAPdenovo version 2.21 (5).

The draft assembly of *C. chlorophyti* consists of 558 scaffolds with a total length of 52.4 Mb (*N*_50_: 644,295; *N*_75_: 313,035; *L*_50_: 26; *L*_75_: 56) and a G+C content of 50.06%. The completeness of the assembly was assessed using a set of 1,438 conserved fungal genes identified as benchmarking universal single-copy orthologs using the BUSCO version 1.1b1 program (6). From this analysis, the assembly was estimated to include 99.9% of the assessed loci (98.5% complete, 1.3% fragmented).

Protein-coding genes were predicted using the MAKER release 2.31.8 (5) annotation pipeline with Augustus version 3.1 (7), GeneMark-ES version 4.21 (8), and SNAP (9) with conserved proteins from the genome of *C. incanum* (10) as a training set. Augustus was trained using a set of *C. chlorophyti* genes identified using the CEGMA set of conserved eukaryotic genes identified with CEGMA version 2.5 (11). A total of 10,419 protein-coding genes were predicted in the genome. Predicted proteins were classified as secreted when predicted to have a signal peptide using SignalP version 4.1 (12), to have no transmembrane domains according to TMHMM version 2.0 (13), and to have no GPI anchors according to BIG-PI fungal predictor (14). Gene-coding sequences were annotated with the Trinotate version 3.0.0 program (https://trinotate.github.io) by integrating information from the SWISS-PROT (15) and Pfam (16) databases. A total of 851 proteins were predicted to be secreted, including 279 that had no match in the Swissprot (15) database.

### Accession number(s).

The sequences were deposited in DDBJ/EMBL/GenBank under the accession number MPGH00000000. The version described in this paper is the first version, MPGH01000000. Files are also available at: https://sites.google.com/site/colletotrichumgenome.

## References

[1] Damm U, Woudenberg JHC, Cannon PF, Crous PW 2009 *Colletotrichum* species with curved conidia from herbaceous hosts. Fungal Divers 39:45–87.

[2] Yang H-C, Stewart JM, Hartman GL 2013 First report of *Colletotrichum chlorophyti* infecting soybean seed in Arkansas, United States. Plant Dis 97:1510–1510. doi:10.1094/PDIS-04-13-0441-PDN. 30708473

[3] Cannon PF, Damm U, Johnston PR, Weir BS 2012 *Colletotrichum*—current status and future directions. Stud Mycol 73:181–213. doi:10.3114/sim0014. 23136460 PMC3458418

[4] Bolger AM, Lohse M, Usadel B 2014 Trimmomatic: a flexible trimmer for Illumina sequence data. Bioinformatics 30:2114–2120. doi:10.1093/bioinformatics/btu170. 24695404 PMC4103590

[5] Luo R, Liu B, Xie Y, Li Z, Huang W, Yuan J, He G, Chen Y, Pan Q, Liu Y, Tang J, Wu G, Zhang H, Shi Y, Liu Y, Yu C, Wang B, Lu Y, Han C, Cheung DW, Yiu SM, Peng S, Xiaoqian Z, Liu G, Liao X, Li Y, Yang H, Wang J, Lam TW, Wang J 2012 SOAPdenovo2: an empirically improved memory-efficient short-read de novo assembler. GigaScience 1:18. doi:10.1186/2047-217X-1-18. 23587118 PMC3626529

[6] Simão FA, Waterhouse RM, Ioannidis P, Kriventseva EV, Zdobnov EM 2015 BUSCO: assessing genome assembly and annotation completeness with single-copy orthologs. Bioinformatics 31:3210–3212. doi:10.1093/bioinformatics/btv351. 26059717

[7] Stanke M, Schöffmann O, Morgenstern B, Waack S 2006 Gene prediction in eukaryotes with a generalized hidden Markov model that uses hints from external sources. BMC Bioinformatics 7:62. doi:10.1186/1471-2105-7-62. 16469098 PMC1409804

[8] Lomsadze A, Burns PD, Borodovsky M 2014 Integration of mapped RNA-Seq reads into automatic training of eukaryotic gene finding algorithm. Nucleic Acids Res 42:e119. doi:10.1093/nar/gku557. 24990371 PMC4150757

[9] Korf I 2004 Gene finding in novel genomes. BMC Bioinformatics 5:59. doi:10.1186/1471-2105-5-59. 15144565 PMC421630

[10] Gan P, Narusaka M, Kumakura N, Tsushima A, Takano Y, Narusaka Y, Shirasu K 2016 Genus-wide comparative genome analyses of *Colletotrichum* species reveal specific gene family losses and gains during adaptation to specific infection lifestyles. Genome Biol Evol 8:1467–1481. doi:10.1093/gbe/evw089. 27189990 PMC4898803

[11] Parra G, Bradnam K, Korf I 2007 CEGMA: a pipeline to accurately annotate core genes in eukaryotic genomes. Bioinformatics 23:1061–1067. doi:10.1093/bioinformatics/btm071. 17332020

[12] Petersen TN, Brunak S, von Heijne G, Nielsen H 2011 SignalP 4.0: discriminating signal peptides from transmembrane regions. Nat Methods 8:785–786. doi:10.1038/nmeth.1701. 21959131

[13] Krogh A, Larsson B, von Heijne G, Sonnhammer EL 2001 Predicting transmembrane protein topology with a hidden Markov model: application to complete genomes. J Mol Biol 305:567–580. doi:10.1006/jmbi.2000.4315. 11152613

[14] Eisenhaber B, Schneider G, Wildpaner M, Eisenhaber F 2004 A sensitive predictor for potential GPI lipid modification sites in fungal protein sequences and its application to genome-wide studies for Aspergillus nidulans, *Candida albicans*, *Neurospora crassa*, *Saccharomyces cerevisiae* and *Schizosaccharomyces pombe*. J Mol Biol 337:243–253. doi:10.1016/j.jmb.2004.01.025. 15003443

[15] Bairoch A, Apweiler R 2000 The SWISS-PROT protein sequence database and its supplement TrEMBL in 2000. Nucleic Acids Res 28:45–48. doi:10.1093/nar/28.1.45. 10592178 PMC102476

[16] Finn RD, Bateman A, Clements J, Coggill P, Eberhardt RY, Eddy SR, Heger A, Hetherington K, Holm L, Mistry J, Sonnhammer ELL, Tate J, Punta M 2014 Pfam: the protein families database. Nucleic Acids Res 42:D222–D230. doi:10.1093/nar/gkt1223. 24288371 PMC3965110

